# Calvarial Tuberculosis in Patient With Cervical Tuberculous Lymphadenitis: A Case Report

**DOI:** 10.7759/cureus.47055

**Published:** 2023-10-15

**Authors:** Rawan Almutairi

**Affiliations:** 1 Infectious Disease, Infectious Disease Hospital, Andalous, KWT

**Keywords:** tb osteomyelitis, head and neck radiology, cervical tuberculous lymphadenitis, tuberculosis, calvarial tuberculosis

## Abstract

Calvarial tuberculosis (TB) is an uncommon form of TB reported in patients with mycobacterial infections. We present a case of calvarial TB in a patient with cervical TB lymphadenitis. The patient had a history of headache and swelling of the right parietal region of the skull. CT head showed peripherally enhancing small epidural collection at the right parietal region with overlying destroyed right parietal bone. Histopathology showed giant cells, lymphocytes, and caseous necrosis. We acknowledge that cervical TB lymphadenitis poses the development of calvarial TB in our patient.

## Introduction

TB is an infectious disease caused by *Mycobacterium tuberculosis* that mainly involves the lungs, with pulmonary disease being the most common manifestation. It is a multisystemic disease that can affect a variety of organs. This includes the digestive system, lymph nodes, skin, central nervous system, musculoskeletal system, reproductive system, liver, and spleen [1,2]. Calvarial TB is an uncommon form of TB, reported in less than 0.01% of patients with mycobacterial infections [3]. Herein, we present a case of calvarial tuberculosis in a patient with cervical tuberculous lymphadenitis.

## Case presentation

A 52-year-old female patient, who is currently undergoing treatment for tuberculosis, presented with a one-month history of headache and a gradual increase in swelling across the right parietal region of the skull over the course of two weeks. Headache severity increased over the past week and was localized to the right parietal region without radiation. The patient also experienced intermittent fever, without chills or rigor. The patient had no history of trauma or weight loss. The patient was neither a smoker nor an alcoholic, and she reported no respiratory symptoms.

Two weeks before the headache, she was diagnosed with cervical TB lymphadenitis by ultrasound-guided fine needle aspiration cytology, which was positive for acid-fast bacilli, upon her presentation with multiple cervical lymph node enlargements and three neck swellings, and managed with incision and drainage with anti-TB medications. She was also infected with pulmonary TB in Bangladesh 25 years ago and received a full course of anti-TB medications.

On examination, the dimension of the fluctuating scalp swelling lesion was 2×1.5 cm, it was nonpulsatile, soft, and its borders were unclear. A small defect was palpable in the parietal region, with a small amount of discharged pus. Multiple lymph node enlargements were also observed in the cervical region. No sensory-motor deficit and cranial nerves were intact. There were no signs of meningeal irritation, and the plantar reflex was downward bilaterally.

**Figure 1:**
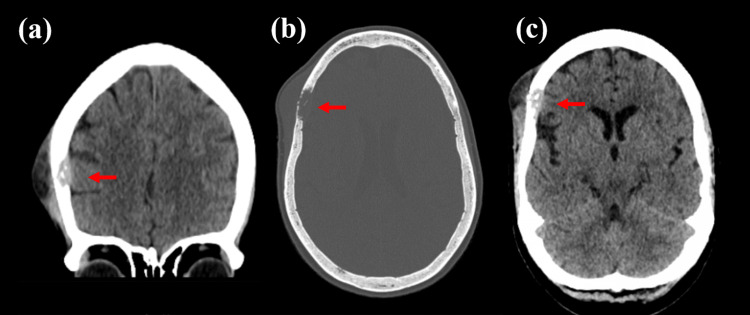
Computed tomographic scan of the head showed calvarial TB. (a) Coronal CT scan demonstrates a peripherally enhancing epidural collection at the right parietal region with overlying destructed right parietal bone not crossing the suture and sub-galeal lesion. (b) Axial bone window demonstrates the disintegration of the right parietal bone. (c) Axial CT image demonstrating the outer and interior table defect with extradural collection.

Laboratory investigations revealed no leukocytosis and the erythrocyte sedimentation rate was normal. HBA1C was normal. The chest radiograph was unremarkable. Fine needle aspiration of the scalp pus was negative for bacteria, fungus, and acid-fast bacilli. Histopathological examination of the inflamed peri-cranium showed giant cells, lymphocytes, and caseous necrosis, along with bone fragments. The Mantoux test result was negative. CT head showed an enhancing small epidural collection at the right parietal region with a destructed right parietal bone associated with a sub-galeal lesion (Figures 1a-1c). The patient was admitted and managed with debridement and continued anti-TB medications.

## Discussion

Skeletal TB is observed in approximately 1% of patients with mycobacterial infections. Among these cases, the prevalence of skull bone tuberculosis is estimated to range from 0.1% to 3.7%, indicating that it is a very uncommon disease globally [4]. Approximately 75-90% of those diagnosed with calvarial tuberculosis fall within the age range of 20-30 years [5].

Reid reported the first case of calvarial tuberculosis in 1842 [6]. It has been observed to affect both sexes equally, with no sex predilection [7]. Although TB osteomyelitis can develop in any bone of the skull vault, frontal and parietal bones are the most commonly involved sites [8]. This is probably due to the fact that these cranial bones have a greater amount of cancellous bone compared with other cranial bones [5]. Our patient also showed involvement of the parietal bone of the skull.

Calvarial TB occurs in most patients secondary to TB elsewhere in the body, with the highest frequency in the lungs. After damage by one or more granulomas of TB in any body part, TB bacteria enter the diploe of the cranial bones via circulation. Despite this, some authors have proposed that TB bacteria can spread to the bones of the skull vault via lymphatic vessels. This would also explain the rarity of calvarial TB, as the skull has a weak lymphatic supply [9]. We acknowledge that cervical TB lymphadenitis poses the development of calvarial TB in our patient.

The presentation of calvarial TB varies significantly. The most common presentations are fluctuating scalp swelling and discharging sinus. Other rare presentations include low-grade fever, headaches, seizures, and other neurological deficits [10,11]. In our case, swelling, headache, and occasional fever were the only symptoms that were observed.

CT of the head shows swelling of the soft tissues with disintegration of the inner and/or outer skull tables. A skeletal sequestrum can also be observed. It also reveals that the disease has spread to the extradural space, meninges, and brain parenchyma. Granulation tissue of the epidura appears as crescentic or lentiform low-attenuation collections. After administration of a contrast medium, the adjacent meninges enhance greatly. In addition, the CT scan revealed signs of meningitis and parenchymal diseases [5].

Detection of acid-fast bacilli in pus smears by Ziehl-Neelsen staining or isolation by culture is crucial in diagnosing the disease. In addition, the diagnosis is determined by histopathological findings with multiple epithelioid granulomas and necrotic material. The following tests were negative in our patient, including Mantoux, interferon-gamma release assay (IGRA), and the detection of acid-fast bacilli. We believe that this was a result of taking anti-TB medications for two weeks. Moreover, it has been shown that 46% of patients with active TB have negative IGRA results with anti-TB treatment [12,13]. Our case was diagnosed based on clinical findings, radiological characteristics, and histology.

Calvarial TB is managed with surgery and a full course of anti-TB medications. Surgery is indicated for drainage of abscesses and lesions causing mass effects and neurological impairments [14].

## Conclusions

Calvarial TB is a rare form of skull tuberculosis. It is believed to occur by hematogenous seeding of bacilli to the diploe or by lymphatic dissemination of tuberculosis. It is usually secondary to a pulmonary origin and can also be associated with cervical TB lymphadenitis. Common radiological findings include soft tissue swelling and destruction of one or both skull tables. Patients respond well to antitubercular medications and surgical drainage if needed.
